# The natural course of low back pain: a systematic critical literature review

**DOI:** 10.1186/2045-709X-20-33

**Published:** 2012-10-17

**Authors:** Nadège Lemeunier, Charlotte Leboeuf-Yde, Olivier Gagey

**Affiliations:** 1 Complexité, Innovation et Activités Motrices et Sportives, Bâtiment 335, UFR STAPS, Université Paris Sud-11, Orsay Cedex, 91405, France; 2 Institut Franco-Européen de Chiropratique, 72 Chemin de la Flambère, Toulouse, 31300, France; 3 Research Department, Spine Center of Southern Denmark, Hospital Lillebaelt and Institute of Regional Health Services Research, University of Southern Denmark, Ostre Hougvej 55, Middelfart, Denmark

**Keywords:** Low back pain, Course, General population, Prospective study, Review

## Abstract

**Background:**

Most patients in the secondary care sector consulting for low back pain (LBP) seem to have a more or less constant course of pain during the ensuing year. Fewer patients with LBP in the primary care sector report continual pain over a one-year period. However, not much is known about the long-term course of LBP in the general population. A systematic critical literature review was undertaken in order to study the natural course of LBP over time in the general population.

**Methods:**

A search of articles was performed in Pubmed, Cinahl and Psychinfo using the search terms ‘epidemiology’; ‘low back pain’ or ‘back pain’; ‘prospective study’ or ‘longitudinal study’; ‘follow-up’, ‘natural course’, ‘course’ or ‘natural history’; ‘general population’ or ‘working population’. Inclusion criteria were that one of the objectives was to study the course of (L)BP in the adult population, that the period of follow-up was at least 3 months, and that there were three points of observation or more. The review was undertaken by two independent reviewers using three checklists relating to description of studies, quality and outcomes. The course of LBP was established in relation to those who, at baseline, were reported not to have LBP or to have LBP. Would this course be stable, fluctuating, worsening, or improving over time? A synthesis of results in relation to common patterns was presented in a table and interpreted in a narrative form.

**Results:**

Eight articles were included. Articles were different on time span, the number of surveys, and the definition of LBP. In six of the seven relevant studies, for those with no LBP at baseline, relatively substantial stable subgroups of people who continued to be LBP free were identified. In six of the seven relevant studies, definite stable subgroups of continued LBP were noted and improvement (becoming pain free) was never reported to be a common finding.

**Conclusion:**

The status of LBP in individuals of the general population appears to be relatively stable over time, perhaps particularly so for those without LBP at baseline.

## Introduction

Low back pain (LBP), which is a common disorder in the general population, was previously considered a generally short lasting disease with spontaneous recovery the most likely outcome. Because it is difficult to provide specific diagnoses to this condition, it became common to classify it according to the duration of the pain (i.e. acute, subacute or chronic) [1] with chronicity being considered relatively uncommon [2]. Nowadays, LBP is considered, rather, to be a recurring or persistent condition with a fluctuating course over time [3,4]. Yet, patients in the secondary care sector consulting for LBP seem to have a more or less constant course of pain during the ensuing year [5]. In contrast, fewer patients with LBP in the primary care sector report continual pain over a one-year period [6]. However, not much is known about the long-term course and different course patterns of LBP in the general population.

Our objective was to conduct a systematic critical literature review to improve our understanding of the natural course of LBP in the general population. Specifically, we wanted to find out the proportions of people with LBP at baseline who, when studied over prolonged periods of time, got better, worse, remained unchanged or fluctuated between LBP and no LBP. Similarly, we wanted to identify the most common course patterns for people without LBP at baseline; would they remain LBP free, develop LBP over time, or fluctuate between LBP and no LBP?

## Method

### Search strategy

A search for articles was performed in Pubmed, Cinahl and Psychinfo (until May 2012) using the search terms: ‘epidemiology’; ‘low back pain’ or ‘back pain’; ‘prospective study’ or ‘longitudinal study’; ‘follow-up’, ‘natural course’, ‘course’ or ‘natural history’; ‘general population’ or ‘working population’. None of our two universities had access to Embase. An additional citation search was performed of reference lists of the retrieved articles. We used no restrictions for date of publication or language.

### Inclusion and exclusion criteria

Selection of articles were made by NL and verified by CLY according to predetermined criteria for inclusion and exclusion that were defined by NL and CLY in relation to the objectives of the review.

Articles were included if (one of) their stated objective(s) was to study the course of (L)BP in the general population, i.e. no studies concerning clinical populations or pregnancy were included. Further inclusion criteria were that LBP should be measured at baseline and at a minimum of 2 subsequent follow-ups. Studies should report on the same individuals (n > 100) for a minimum of 3 months and participants should be ≥ 18 years old.

Because we expected to find only few studies of the general population, we also included studies of specific working populations if they did not represent hard physical work (e.g. construction workers) or extreme postures (e.g. painters, vineyard workers). Studies from the army or on compensation cases were also not accepted.

### Checklists

Three checklists were created especially for this subject. These checklists related to the description of studies (Additional file 1: Appendix 1), their methodological quality (Additional file 1: Appendix 2), and their results. The criteria for methodological quality have been listed under three main headings: 1) representativeness of the study sample, 2) quality of data and 3) clear definition of LBP (Additional file 1: Appendix 2). The quality grid consisted of a slightly modified list of items previously used for prevalence studies of LBP [7]. All check-lists were tested by the reviewers for relevance and user friendliness on two articles, modified as needed to fit the purpose of the review, and tested once more prior to use.

### Review process and interpretation of findings

Each article that fitted the criteria was independently and blindly reviewed by NL and CLY. In case of disagreement, the third author would be consulted. For all studies, the presence or absence of criteria was noted and the response rates were sought out or, if necessary, calculated. Only information mentioned in the methods or results sections was taken into account. A quality score was then calculated for each article according to the total number of acceptable criteria divided by the total number of relevant criteria. Each article was scrutinized for methodological quality, using the previously described scoring system but without determining an à priori cut-point for a minimal score, using it as an informative rather than a prescriptive score.

Results were taken into account only in relation to the pain aspect such as presence of LBP, duration, severity, or pattern; i.e. disability and consequences of LBP were not considered. The result sections were scrutinized for description of the course over time in relation to those who, at baseline, were reported a) not to have LBP or b) to have LBP. Results in each study were sought out in relation to whether absence or presence of LBP was stable, fluctuating, worsening, or – in the case of those having LBP at baseline -improving over time. The findings were reported in a table and interpreted in a narrative fashion. In addition, results were analyzed in relation to type of population and the number and spacing of surveys.

## Results

### Number of articles

Initially in Pubmed, 18 articles were considered suitable based on their title and study objectives ( Additional file 1: Appendix 3). Of these, only 8 were retained after scrutiny of their text for all inclusion and exclusion criteria [8-15]. The 9 articles found with the search in Cinahl database overlapped with those already found in Pubmed. No relevant articles were found in the Psychinfo database. The additional citation search did not result in any relevant publications. An additional article was found in one of the authors’ archives.

Some discussion between the reviewers was necessary for most articles, not because of disagreement but in order to clarify points that were unclear in the text; particularly in relation to the definition of LBP and the various response rates. There was no need to call in the third author for arbitration.

### Description of the articles

The eight accepted articles had all been published since 1997, reporting on studies having been conducted between 1991 and 2005. Three had been carried out in the Nordic countries (Finland and Denmark), two in Switzerland and the rest in Northern Europe (UK, Netherlands and Germany).

As seen in Table 1, there were four articles on the general population [9,12-14] and four on specific working population (nurses [8,15], hospital employees [10] and employees of factories [11]). One article [12] included only people with previous LBP. In all but one of the reports, participants were between 20 and 60 years, the eight article only provided the mean age with the SD (23.2(5.1) [15]. In no two articles was the duration of the entire study period or the numbers of surveys identical, ranging from 52 surveys over one year [12] to 4 surveys over 28 years [11]. Four of the studies used the Nordic Back Pain Questionnaire either in postal surveys [9,10], through the internet or via postal diaries [12], or by computer assisted telephonic interview [14]. For the remaining four [8,11,13,15], questionnaires of unknown source were used. Although the exact wording of the LBP question was not always the same, definitions of LBP were generally relatively similar (usually LBP in the past year) with only two concentrating on LBP in the past month [8,12]. One reported also on longstanding LBP [14] and another used the description ‘severity’, which we renamed ‘duration’, as it related to number of days in the past year and not severity of symptoms [12]. One of the articles related the recall period to the duration since the last survey [15]. 

**Table 1:** Descriptive checklist

**Reference number**	**I**^**st**^**author Year Country**	**Type of population (Age range)**	**Specific inclusion criteria in relation to LBP**	**Method of data collection**	**Definition of relevant LBP outcome variable (Anatomical site, recall periods, duration, severity, consequence)**	**Years or time of surveys**	**Numbers of surveys over the study period/years**
[8]	Smedley 1998 UK	University hospital-based nurses, all types (19–64 years)	NA	Q^aire^	LBP > 1 day in the past month	1993	8/2
						Every 3 month Until 1995	
[9]	Hestbaek 2003 Denmark	Men and women living in a Danish municipality (30–50 years)	NA	Q^aire^	Number of days with LBP in the past year (0; 1–7; 8–30; >30) days	1991	3/5
						1992	
						1995	
[10]	Maül 2003 Germany	University hospital-based nurses (?)	NA	Q^aire^	Number of days with LBP past year (0; 1–7; ≥8)* days	1991	3/9
						1992	
						1999	
[11]	Kääriä 2006 Finland	The employees in factories, all types (at least 47 yrs)	NA	Q^aire^	LBP in the past year	1973	4/28
						1978	
						1983	
						2000	
[12]	Tamcan 2010 Switzerland	General population (?)	Those who report LBP in 2002–03 and who still report LBP in 2005	Internet-based diaries or postal diaries	LBP past month at week 1 and week 53	2005	53/1
					Intensity of pain each week between	Every week	
							
[13]	Kolb 2011 Switzerland	General population (?)	NA	Computer assisted telephonic interviews	In past year >1 month of bad BP or LBP	1999	5/5
						2000	
						2001	
						2002	
						2003	
[14]	Van Oostrom 2011 The Netherlands	General population (20–60)	NA	Q^aire^	Persistent LBP past year	1993-97	3/10
					Defined as more than three months (study 1 and 3) and more than one month (study 2)	1998-02	
						2003-07	
[15]	Videman 2005 Finlande	Nursing students	NA	Q^aire^	BP past 4 month, past year and past 4 years	Baseline	9/7.5
					(0; 1–7; 8–30; >30) daysbut not daily, and daily	Every 4 month during 2 years	
						1 year after school	
						5 year after school	

*Mentionned in methods. Reclassified from ‘severity’ to duration.

*NA*: Not applicable.

Furthermore, LBP was not always described in the same way between studies. In four articles [8,11,13,15], the presence or absence of LBP in the past year was measured at each survey without further specification; in two articles [9,10], LBP was classified in relation to duration during the past year; one article [12] categorized LBP according to severity and persistence of symptoms, whereas another article [14] used two different definitions for longstanding LBP in the past year (>3 months in two surveys and >1 month in one survey).

### Quality of studies

Table 2 shows that all articles had a fairly high score according to the quality checklist; none scored less than 7/11. For this reason we took no further notice of the quality score, as we considered all articles to be credible. Nevertheless, it is worth noticing that two of the articles did not clearly deal with the issue of representativeness [8,10]. Other quality issues of interest are described below. 

**Table 2:** Quality checklist

**Ref N°**	**Representativeness**					**Quality of data**			**Definition of LBP**			
	**Response rates in relation to invited study sample at baseline.**	**Sample sizes**	**N/% response at all surveys based on number of participants at first survey**	**At least one of the following: Whole target population; Randomly selected sample; or Sample stated to represent general population**	**At least one of the following: Reasons for non response described; Non-responders described; Comparison responders-non-responders; or Comparison of sample and target population**	**Same mode of data collection for all subjects, and all surveys**	**Same definition(s) of LBP outcome variable used for all subjects at all survey**	**At least one of the following: Questionnaires, diaries, or interviews validated; Tested for reproductibility or Tested in pilot study**	**Precise anatomical delineation of lumbar area; or Reference to easily obtainable article that contains such specification**	**Further specification of definition of LBP; Questions put to study subjects quoted; or Reference to easily obtainable article that contains such specification**	**Recall periods specified**	**Quality score: Number of ‘Yes’/Number of relevant items**
	**If new study subjects invited, response rates calculated based on number of invited participants at each survey**			***(Yes/No)***	***(Yes/No)***	***(Yes/No)***	***(Yes/No)***	***(Yes/No)***	***(Yes/No)***	***(Yes/No)***	***(Yes/No)***	
[8]	1088/2405 = 45 %	1088	470/1165	No	Yes	Yes	Yes	No	Yes	Yes	Yes	9/11
	999/2405 = 41%	999	40%									82%
	878/2405 = 36%	878										
	827/2405 = 34%	827										
	758/2405 = 31%	758										
	700/2405 = 29%	700										
	614/2405 = 25%	614										
	599/2405 = 25%	599										
[9]	1309/2000 = 65%	1309	765/1309	Yes	Yes	Yes	Yes	Yes	Yes	Yes	Yes	11/11
	1198/2000 = 60%	1198	58%									100%
	813/2000 = 41%	813										
[10]	1307/1963 = 67%	1307	269/1307	No	Yes	Yes	Yes	Yes	Yes	Yes	Yes	10/11
	1159/2185 = 53%	1159	21%									91%
	1584/2744 = 58%	1584										
[11]	902/1057 = 85%	902	418/902	Yes	No	Yes	Yes	No	No	Yes	Yes	8/11
	748/1057 = 71%	748	46%									73%
	654/1057 = 62%	654										
	546/1057 = 52%	546										
[12]	340/400 = 85%	340	206/340	Yes	Yes	Yes	Yes	Yes	Yes	Yes	Yes	11/11
	participated		76%									100%
	Used in analysis: 305/340 = 90%		(95-100% responses)									
[13]	?	7791	3881/7791	Yes	Yes	Yes	Yes	No	No	Yes	Yes	8/11
		6335	50%									73%
		5755										
		4885										
		4354										
[14]	6118/7769 = 79%	6118	4007/6118	Yes	Yes	Yes	No	Yes	Yes	Yes	Yes	10/11
	4917/7769 = 63%	4917	65%									91%
	4520/7769 = 58%	4520										
[15]	?/308	?	108/308	No	Yes	Yes	Yes	No	Yes	Yes	Yes	7/11
	?/308	?										64%
	?/308	?										
	?/308	?										
	?/308	?										
	?/308	?										
	?/308	?										
	?/308	?										
	197/308	197										
	174/308	174										

Not all reported the response rate in percentages [8,10-12,15] but when reported these ranged from 34% [13] to 96% [9]. In the article in which data were collected 52 times (every week) during one year [12], participants who completed at least 50% of these questionnaires were defined as ‘responders’, resulting in a total response rate of 90%. However, as is often the case in prospective studies, not everybody reported response rates for each subsequent survey clearly in relation to either those invited to participate in the first survey or (if that number was unknown) at least in relation to the number of participants at baseline. A calculation based on these figures reduced the response rates to a range from 21% [10] to 65% [14]. Furthermore, only five reports [9-11,14,15] discussed the potential impact that the non-responders may have on the results and in only one article were data modeled for this group [9].

### Course of LBP

Table 3 gives the results on the course of absence or presence of LBP for each article. Interpretation of the natural course of LBP is reported below both for those without and those with LBP at baseline, in relation to stability, fluctuation, worsening or improvement.

**Table 3:** Results

**At Baseline**	**Reference N°**	**Development of LBP over time**				**Comments**
		**Stable**	**Fluctuating**	**Worsening**	**Improving**	
**No LBP**	[8]	No LBP at BL was highly predictive of future absence of pain throughout 8 surveys over 2 years			NA	
	[9]	45% with no pain at 3 surveys over 5 years			NA	
	[10]	70% no pain at second survey and 57% at 3^rd^ survey over 8 years			NA	
	[11]	67% and63% respectively no pain at 2^nd^ and 3^rd^ survey over 28 years		64% had LBP at the 4^th^ survey (28 years later)	NA	
	[12]	NA	NA	NA	NA	Not applicable: all participants were chosen because they had LBP
	[13]	The most frequent course was no BP each year over 5 years (35%)			NA	
	[14]	- 29% of the population was free of LBP at 3 surveys over 10 years		- 11% developed long standing LBP at 2^nd^ and 3^rd^ survey over 10 years	NA	
		- 62% never had long standing LBP at 3 surveys over 10 years				
	[15]	Stable (visual analysis)				
**Presence of LBP**	[8]	Presence of LBP at BL was highly predictive of future pain throughout 8 surveys over 2 years				
	[9]	If >30 days of LBP at BL: 39% in the same category after 1 and 5 years	If 1–30 days of LBP at BL: 62% fluctuated to the neighboring groups over 5 years			
	[10]	38% have the same intensity of LBP at 3 surveys over 8 years	27% of LBP (intensity) fluctuated; movements between extremes groups were rare (12%) at 3 surveys over 8 years	17% of LBP (intensity) increased at 3 surveys over 8 years	19% of LBP (intensity) decreased at 3 surveys over 8 years	
	[11]	75, 73 and 88% were symptomatic at 3 FUs over 28 years				
		31% of the subjects reported LBP in all 4 surveys				
	[12]	Stability of severity and frequency of LBP was high in 4 periods over 1 year			3% reported no pain after BL throughout the weekly surveys over one year	
	[13]	The most frequent course was BP each year over 5 years (14%)				
	[14]	6% had long standing LBP at 3 surveys over 10 years	11% had long standing LBP only at some surveys over 10 years		10% had recovered from long lasting low back pain at 2^nd^ and 3^rd^ survey	
	[15]	For those who had more than 8 days of BP during that first year (visual analysis)	For those who have between 1–7 days of BP the first year of the 5-yr study period (visual analysis)			

*LBP*: Low back pain; *FU*: Follow-up; *BL*: Baseline; *NA*: Not Applicable.

#### ***No LBP at baseline***

As shown in Table 3, in six of the seven relevant studies, relatively substantial stable stable subgroups were identified of people who continued to be LBP free. In one study [8], absence of LBP at baseline was said to be predictive of continued absence of LBP. In another study [13], absence of LBP was noted to be the most common subgroup of 32 possible combinations and in another [9], almost 50% belonged to this category. According to one of these six studies, approximately 10% with no LBP at baseline reported long standing LBP five and ten years later [14]. Further, at the 28 years follow-up, LBP was reported by 2/3 of those initially free of LBP [11].

#### ***LBP at baseline***

The course over time in those who reported LBP at baseline seemed to be somewhat more heterogeneous (Table 3). In all of the seven relevant studies, definite stable subgroups of continued LBP were noted and improvement (becoming pain free) was never reported to be a common finding. According to one article [11], LBP was a stable occurrence five, ten and 28 years down the track, and also when surveyed weekly over one year [12], persistence of symptoms was noted in the majority of participants.

When fluctuation occurred (n = 4), it seemed most common between neighboring groups [9,10,14,15]. One study identified also a relatively small subgroup of people that worsened over time [10].

### Additional analyses

There were no obvious differences in our results in relation to type of study population or number and spacing of surveys, with the possible exception of the results for the 28-year follow-up that indicated that LBP will occur in the end among the previously ‘protected’ non-sufferers [11].

## Discussion

The purpose of this review was to gain an understanding of the natural course of LBP. The conclusion is that the LBP status at baseline is predictive of the future course and, probably, in particular for those who do not have LBP at baseline.

The eight studies that were identified were all of relatively high quality, judging by their quality scores, but their study approaches were dissimilar in relation to definition of LBP, method of data collection, number of surveys, time between surveys, and type of population. That the results nevertheless pointed in the same direction, strengthens the validity of these findings. However, it would have been helpful if studies could have reported their data more clearly and systematically, as otherwise it is difficult to extract the relevant information from the text.

In particular, it would have been more informative if researchers could have reported more clearly the percentage of drop-outs at the various surveys and attempted to take into account the possible effect of missing data. Although several authors [9-11,14,15] considered potential differences between responders and non-responders, only one [9] visualized them in their result section and even took them into account in a best case and worst case analysis, which obviously can be important in studies with large dropout rate numbers, as is often the case in studies with multiple follow-ups over long periods of time.

This systematic and critical review was done independently by two readers with no particular interest in the outcome of the review. Nonetheless, it suffers the same potential weaknesses as many other similar reviews. For example, it is not sure that all relevant articles were retrieved, if checklists were relevant, or if the information was properly interpreted.

However, this topic is fairly new, indicating that there would not be numerous studies and those that have been published were easily noticed. Further, our thorough citation search did not result in any additional publications, although, admittedly, one retrieved article [15] failed to be captured in our search procedure. Still, it is possible that this type of data can be found interspersed between the main messages of articles with other specific objectives than describing the natural course of LBP. It is possible that we may have missed those. The grids for systematic data collection were designed to meet our needs and the quality checklist was a previously published and used checklist for this type of studies with only minor adjustments to fulfill the needs of the present review. Another type of quality checklist could of course have resulted in a different view of which articles to accept for analysis. Although the literature sometimes was difficult to extract and interpret, partly because not all articles had the same primary research objectives as we had, it was never necessary to seek arbitration from the third author, indicating good consensus between reviewers although, of course, not guaranteeing accuracy.

Another potential shortcoming could be that we included studies also from the working population. Such studies could have biased study samples either through a healthy worker effect [16] or the opposite, in the case of physically undemanding jobs. In our case, a healthy worker effect would probably not be pronounced, as we on purpose did not include working populations representing heavy manual labor. Also, there were no obvious differences in outcomes between studies of the general and working populations.

## Conclusion

The results of this survey indicate that, in the general population, absence of LBP at one time in life is a blessing, in that it will indicate also a pain-free future at last for a fairly large number of years. On the other hand, those with LBP will fairly consistently report LBP again.

## Competing interests

One of the authors (CLY) was co-author on one of the reviewed articles. However, the review was performed by two of the authors, and there were no discrepancies between these two on the findings from that article. Otherwise, the authors state that they have no competing interest.

## Authors’ contributions

All authors helped to plan the review. NL and CLY performed the literature review. NL and CLY interpreted the findings. NL wrote the first draft. All authors participated in completing the manuscript. All authors read and approved the final manuscript.

## Supplementary Material

Additional file 1 **Appendices. 1:** Descriptive checklist for a systematic literature review on the natural course of low back pain (LBP). **2:** Quality checklist for a systematic literature review on the natural course of LBP. **3:** List of 10 articles that were excluded from the literature review in concordance with our inclusion and exclusion criteria. Click here for file
