# Supplementary material for: The natural course of low back pain: a systematic critical literature review
Source: Chiropr Man Therap. 2012 Oct 17;20:33. doi: 10.1186/2045-709X-20-33 (PMC3599187; doi:10.1186/2045-709X-20-33)
Supplement: Additional file 1 — Appendices. 1: Descriptive checklist for a systematic literature review on the natural course of low back pain (LBP). 2: Quality checklist for a systematic literature review on the natural course of LBP. 3: List of 10 articles that were excluded from the literature review in concordance with our inclusion and exclusion criteria. [file 2045-709X-20-33-S1.docx]

**Appendix 1:** Descriptive checklist for a systematic literature review on the natural course of low back pain (LBP):

The following items were used:

1. First author, year of publication and country of study
2. Type of population (general or working population) and age range of participants
3. Specific inclusion criteria in relation to LBP
4. Method of data collection (questionnaire survey, internet or postal diaries, or telephonic computer assisted interviews)
5. Definition of relevant LBP outcome variable (anatomical site, recall period, duration, severity, consequences)
6. Years of each survey
7. Numbers of surveys over the study periods/years

**Appendix 2:** Quality checklist for a systematic literature review on the natural course of LBP

The quality checklist consisted of the following items:

1. The criteria for being able to determine whether the study sample was representative of its target population were:
   - Response rates had to be clearly stated or should be calculated at each point of data collection and this response rate should be possible to calculate on the basis of the number of invited participants at baseline.
   - Sample sizes should be clearly reported for each point of data collection.
   - The presence of at least one of the following: Whole target population, randomly selected sample, or sample stated to represent general population. (Yes/No)
   - The presence of at least one of the following: Reasons for no response described, non-responders described, comparison responders vs. non-responders, or comparison of study sample vs. target population. (Yes/No)
2. The criteria relating to the quality of data were:
   - The data on LBP should have been collected in the same way for all subjects and at each point of data collection. (Yes/No)
   - Identical definition(s) of the LBP outcome variable(s) should have been used for all participants at all points of data collection (Yes/No)
   - At least one of the following: Questionnaires, diaries, or interviews should have been validated, tested for reproducibility, or tested in pilot study (Yes/No). The Nordic Back Questionnaire in its original or modified version was considered to be valid.
3. The criteria relating to the definition of LBP were:
   - Precise anatomical delineation of lumbar area or reference to easily obtainable article that contains such specification (Yes/No)
   - Further specification of definition of LBP, questions put to study subjects quoted, or reference to easily obtainable article that contains such specification (Yes/No)
   - Recall periods specified (Yes/No).

**Appendix 3:** List of 10 articles that were excluded from the literature review in concordance with our inclusion and exclusion criteria [[1-17](#_ENREF_1)]

1. IJzelenberg W and Burdorf A: **Patterns of care for low back pain in a working population.** *Spine* 2004, **29**(12): 1362-8.

2. Mortimer M, Pernold G, and Wiktorin C: **Low back pain in the general population. Natural course and influence of physical exercise. A 5 year follow-up of the Musculoskeletal Intervention Center-Norrtalje Study**. *Spine* 2006, **31**(26): 3045-51.

3. van den Heuvel SG,Ariëns GA, Boshuizen HC, Hoogendoorn WE, Bongers PM: **Prognostic factors related to recurrent low-back pain and sickness absence.** *Scand J Work Environ* *Health* 2004, **30**(6): 459-67.

4. Cassidy JD, Côte P, Carroll LJ, Kristman V: **Incidence and course of low back pain episodes in the general population**. *Spine* 2005, **30**(24): 2817-23.

5. Jacob T: **Low back pain incident episodes: a community-based study.** *The Spine J.* 2006, **6**: 306-10.

6. Clemon G: **The six-month incidence of clinically significant low back pain in the Sakatchewan Adult Population**. *Spine* 2002, **27**(16): 1778-82.

7. Waxman R, Tennant A, and Helliwell P: **A prospective follow-up study of low back pain in the community**. *Spine* 2000, **25**(16): 2085-90.

8. Jacob T, Baras M, Zeev A, Epstein L: **A longitudinal, community-based study of low back pain outcomes**. *Spine* 2004, **29**(16): 1810-7.

9. Müller CF, Monrad T, Biering-Sorensen F, Darre E, Deis A, Kryger P : **The influence of previous low back trouble, general health, and working conditions on future sick-listing because of low back trouble.** *Spine* 1999, **24**(15): 1562-70.

10. Demmelmaier I, Åsenlöf P and Lindberg P: **Biopsychosocial predictors of pain, disability, health care consumption, and sick leave in first-episode and long-term back pain: A longitudinal study in the general population**. *Int J. Behav. med* 2010, **17**: 79-89.
